# Varicella-Zoster Meningitis and Myelitis After Herpes Zoster Dermatitis Treatment With Amenamevir: A Case Series and Literature Review

**DOI:** 10.7759/cureus.54775

**Published:** 2024-02-23

**Authors:** Satoru Tada, Yuta Kaito, Akihiro Watanabe, Yukio Sugiyama, Akira Nishigaichi, Takashi Miwa, Kotaro Watanabe, Takanori Hazama, Daisuke Takahashi

**Affiliations:** 1 Department of Neurology, Osaka University Graduate School of Medicine, Osaka, JPN; 2 Department of Neurology, National Hospital Organization Osaka Minami Medical Center, Osaka, JPN; 3 Department of Rheumatology, National Hospital Organization Osaka Minami Medical Center, Osaka, JPN

**Keywords:** acyclovir, amenamevir, myelitis, meningitis, central nervous system, varicella zoster virus, herpes zoster

## Abstract

Varicella-zoster virus (VZV), known for causing chickenpox, establishes latent infections in neural tissues. Reactivation of VZV can lead to herpes zoster (HZ) and various neurological complications. In this report, we present four cases of VZV meningitis and myelitis following amenamevir treatment for HZ dermatitis with positive VZV DNA in cerebrospinal fluid (CSF) revealed by polymerase chain reaction (PCR). Three of them were considered immunocompromised hosts given the fact that two of these patients were taking immunosuppressive drugs for rheumatoid arthritis, and one patient had a history of sigmoid colon cancer (four months after resection). After HZ onset, amenamevir, which has poor CSF transfer, was prescribed for all the patients, and all of them developed central nervous complications by VZV (meningitis in three cases and myelitis in one case) confirmed by PCR. All the patients were treated with acyclovir, which has a higher CSF transfer, and fully recovered. We speculate that amenamevir might have failed to prevent VZV infection in the central nervous system (CNS) and think that consideration should be given to administering acyclovir in preference to amenamevir for ΗΖ patients at high risk of CNS VZV infection, such as immunocompromised hosts.

## Introduction

Varicella-zoster virus (VZV) is a common viral pathogen that typically manifests as chickenpox in young children. Following the primary infection, VZV establishes a latent presence in the cranial nerves, dorsal root, and autonomic ganglia. However, VZV reactivation can occur in individuals with attenuated VZV-specific T-cell immunity, as often occurs with age. This reactivation can lead to a variety of complications, including herpes zoster (HZ), VZV meningitis, and various neurological symptoms.

Here, we report four cases of aseptic meningitis and viral myelitis following treatment of HZ dermatitis with amenamevir. We also discuss the need for antiviral drug selection, especially in high-risk patients with central nervous system (CNS) complications, because several antiviral drugs have poor CNS penetration. Finally, we discuss the clinical characteristics of amenamevir and the treatment of aseptic meningitis and viral myelitis using cases of HZ with abnormal neurological findings at our hospital.

## Case presentation

Case 1

A 69-year-old man was admitted to our hospital with a one-day history of headache and a seven-day history of facial rash. He was diagnosed with HZ dermatitis at the previous dermatology clinic two days before his admission to our hospital and was prescribed amenamevir (400 mg/day). On admission, he was alert and well-oriented. The patient had a medical history of sigmoid colon cancer four months earlier. His family history was unremarkable. His dermatologic examination revealed scattered vesicles in the area of the first branch of the right trigeminal nerve.

On examination, his consciousness was clear, blood pressure was 132/102 mmHg, pulse rate was 98 bpm, peripheral oxygen saturation was 98% on room air, and body temperature was 36.2°C. Jolt accentuation was observed with negative neck pain, neck stiffness, Brudzinski’s sign, and Kernig’s sign. There were no other abnormal neurological findings. An ophthalmologic examination revealed no obvious uveitis, although elevated intraocular pressure and impaired perception threshold of the right cornea were observed, suggesting subclinical right intraocular inflammation. Blood tests showed increased C-reactive protein (0.38 mg/dL); his estimated glomerular filtration rate was 59.5 mL/min/1.73 m^2^. The blood cell counts and electrolytes were within the normal range. The CSF was clear, and opening pressure was 180 mmH_2_O; the total cell count was 25 cells/μL, mononuclear cell count was 21 cells/μL (84%), glucose level was 54 mg/dL (blood glucose, 102 mg/dL), protein level was 87 mg/dL, and bacterial culture was negative. Polymerase chain reaction (PCR) for VZV DNA in the cerebrospinal fluid (CSF) was positive.

**Figure 1:**
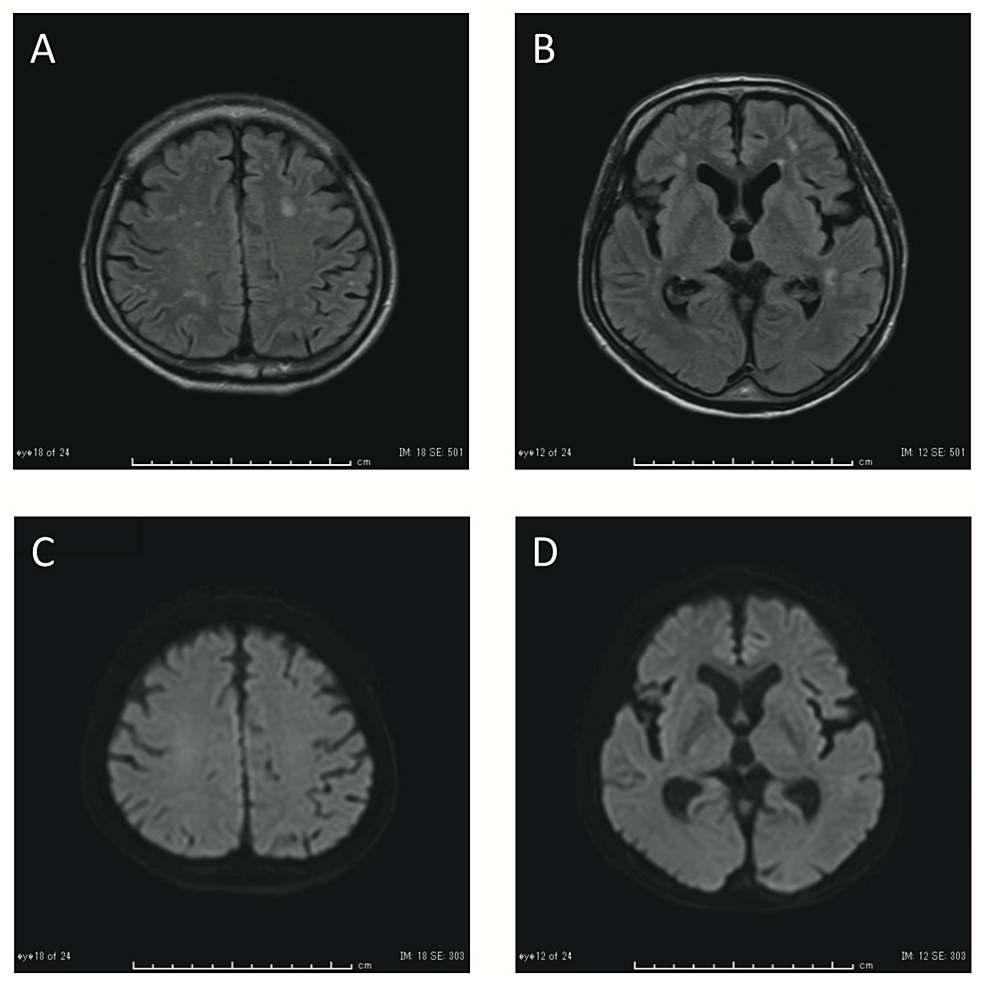
Brain MRI of Case 1 Fluid-attenuated inversion recovery (FLAIR) images (A, B) of the brain MRI showed scattered high-intensity signals in the deep white matter, consistent with old ischemic lesions. Diffusion-weighted images (C, D) showed no acute lesions.

The brain MRI scans did not reveal any acute abnormal findings, which excludes the possibility of vasculitis (Figure 1). Based on the clinical course and biochemical examination, the case was diagnosed as aseptic meningitis complicated by HZ. The patient received treatment with intravenous acyclovir (20 mg/kg body weight/day, 10 days) due to his impaired renal function [1]. By day 5, the headache had resolved, and the blood data showed improved inflammatory findings. On day 10, the patient was discharged without postherpetic neuralgia, HZ recurrence, or meningitis.

Case 2

A 64-year-old woman presented to the outpatient clinic of our hospital with dysesthesia around the left eye and temple of one-day duration. She had a history of rheumatoid arthritis (RA) of five years and renal tuberculosis resulting in left nephrectomy 32 years ago. She was taking salazosulfapyridine 100 mg/day, upadacitinib hydrate 15 mg/day, iguratimod 50 mg/day, and methotrexate (MTX) 6 mg/week. Her family history was unremarkable. Although she did not have any skin lesions around the left eye or in the area of the first branch of the left trigeminal nerve, she was suspected of having HZ based on her subjective symptoms (dysesthesias). She was instructed to stop taking upadacitinib hydrate and MTX and was prescribed amenamevir (400 mg/day) given her history of nephrectomy.

She returned to our outpatient clinic on day 4 with erythema and blistering in the area of the first branch of the left trigeminal nerve, ophthalmalgia, and headache. She was admitted to our hospital on day 5 with a headache and nausea. On admission, she was alert and well-oriented. Her blood pressure was 180/94 mmHg, pulse rate was 76 bpm, peripheral oxygen saturation was 98% on room air, and body temperature was 36.9°C. Jolt accentuation, neck pain, neck stiffness, or Brudzinski’s sign was not observed. There were no other abnormal neurologic findings. Blood tests showed an elevated C-reactive protein (1.23 mg/dL), and her estimated glomerular filtration rate was 57.0 mL/min/1.73 m^2^. Blood counts and electrolytes were normal. The CSF was clear with an opening pressure of 210 mmH_2_O. The total cell count was 9 cells/μL with a mononuclear cell count of 9 cells/μL (100%). The protein level was 50 mg/dL, and the glucose level was 63 mg/dL. Although the bacterial culture was negative, the PCR test for CSF VZV DNA was positive.

**Figure 2:**
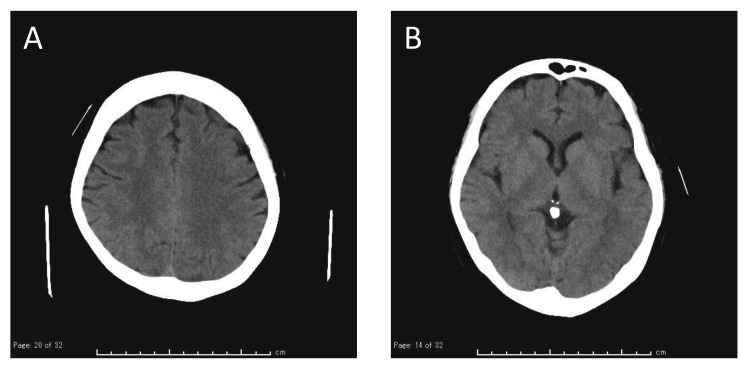
(A-B) Brain CT of Case 2 showing no acute lesions consistent with vasculitis

A brain CT scan showed no acute abnormal findings, thus excluding the possibility of vasculitis (Figure 2). Based on the clinical course and biochemical examination, the case was diagnosed as aseptic meningitis complicated by HZ. The patient was treated with intravenous acyclovir (25 mg/kg body weight/day for seven days), followed by oral acyclovir (3000 mg/day for seven days). Headache and ophthalmalgia were resolved after the administration of pregabalin on day 6. Skin lesions on the face had almost resolved by day 7. She was discharged on day 12 with no neurological complications.

Case 3

A 63-year-old woman was admitted to our hospital with a one-day history of headache, an eight-day history of superficial pain on the left trunk, and a seven-day history of rash on the left flank. She was diagnosed with HZ dermatitis at the previous dermatology clinic six days before her admission to our hospital and was prescribed amenamevir (400 mg/day). On admission, she was alert and well-oriented. She had a history of RA for 18 years and was taking bucillamine (200 mg/day) and prednisolone (PSL, 1 mg/day). A dermatologic examination revealed scattered eruptions on the left flank. Her family history was unremarkable.

On examination, her consciousness was clear, blood pressure was 125/82 mmHg, pulse rate was 80 bpm, and body temperature was 36.7°C. Although she had a headache, jolt accentuation, neck pain, and neck stiffness were not observed, and Brudzinski’s and Kernig’s signs were negative. There were no other abnormal neurological findings. Blood counts and electrolytes were normal. The CSF was clear with the opening pressure of 150 mmH_2_O. The total cell count was 497 cells/μL, with 492 cells/μL (99%) being mononuclear. The protein level was 174 mg/dL, and the glucose level was 44 mg/dL (blood glucose, 101 mg/dL). Although the bacterial culture was negative, PCR analysis for VZV DNA in CSF was positive.

**Figure 3:**
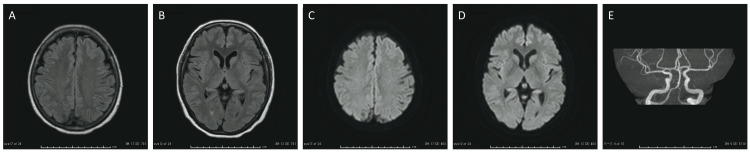
Brain MRI of Case 3 Fluid-attenuated inversion recovery (FLAIR) images (A, B) and diffusion-weighted images (C, D) of the brain MRI showed no old or acute lesions. A magnetic resonance angiography image (E) showed no abnormal findings that were consistent with vasculitis.

Magnetic resonance imaging of the brain did not show any acute abnormalities, thus excluding the possibility of vasculitis (Figure 3). Based on the clinical course and biochemical examination, we diagnosed the case as aseptic meningitis complicated by HZ and administered acyclovir (intravenous infusion, 30 mg/kg body weight/day, 21 days). Acetaminophen was prescribed for the headache, which resolved by day 9, and blood data resulted in improved inflammatory findings. On day 16, the CSF total cell count decreased to 18 cells/μL, and PCR for VZV DNA turned negative, reflecting successful treatment with acyclovir for VZV aseptic meningitis. The patient was discharged on day 23 without postherpetic neuralgia, recurrence of HZ, or meningitis.

Case 4

A 71-year-old man was admitted to our hospital with muscle weakness in both lower extremities for three days and a rash on his left face for eight days. Eight days before admission, he had been diagnosed with HZ dermatitis in his face by his previous dermatologist, who prescribed amenamevir (400 mg/day). At the time of admission, the patient was conscious and well-oriented. He had been suffering from lumbar canal stenosis without surgical intervention for four years. An examination by a dermatologist revealed scattered vesicles in the area of the first and second branches of the left trigeminal nerve on the face. His family history was unremarkable.

On examination, his consciousness was clear; his blood pressure was 120/99 mmHg, pulse rate was 88 bpm, peripheral oxygen saturation was 98% οn room air, and body temperature was 36.9°C. We did not observe jolt accentuation, neck pain, or neck stiffness in this patient. Brudzinski’s and Kernig’s signs were negative.

A neurological examination revealed normal cranial nerves and full strength in neck flexors, neck extensors, and upper extremities. He had 2/5 strength in the iliopsoas, quadriceps, and tibialis anterior bilaterally. Deep tendon reflexes were normal in the upper extremities and increased in the lower extremities. Planter responses were extensor bilaterally, suggesting partial impairment of corticospinal tract integrity. A sensory examination including vibratory, positional, light touch, and temperature sensation and coordination was normal. He was unable to walk due to lower limb weakness. Bladder and rectal dysfunction were not observed.

Blood counts and electrolytes were normal. The CSF was clear; the opening pressure was 160 mmH_2_O, the total cell count was 52 cells/μL, the mononuclear cell count was 42 cells/μL (81%), the protein level was 104 mg/dL, the glucose level was 47 mg/dL (blood glucose, 108 mg/dL), and the bacterial culture was negative. Myelin basic protein (MBP) was 136.5 pg/mL, the IgG index was 0.81, and the CSF oligoclonal band was positive. PCR analysis for VZV DNA in CSF was positive.

**Figure 4:**
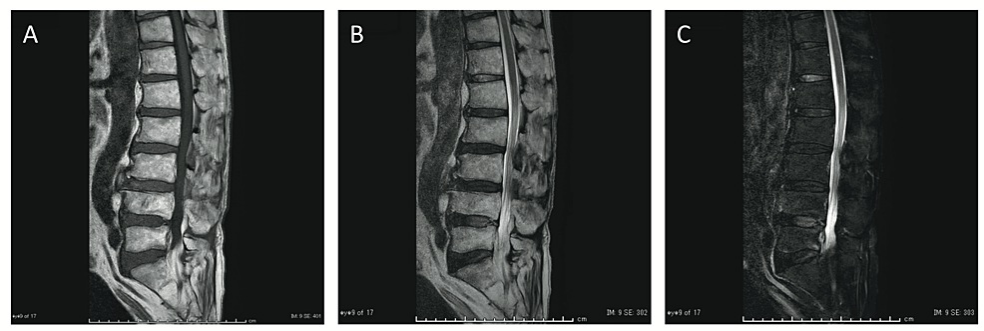
Lumbar spinal cord MRI of Case 4 Τ1-weighted image (A), T2-weighted image (B) and fat-suppressed T2-weighted image (C) of the lumbar spinal cord MRI showed no abnormal findings, including significant canal stenosis, abnormal signal intensity in the spinal cord, or cauda equina.

Brain MRI scans showed no acute abnormal findings, ruling out the possibility of vasculitis. MRI scans of the lumbar spinal cord showed no abnormal findings, including significant canal stenosis, abnormal signal intensity in the lumbar spinal cord, or cauda equina (Figure 4). We diagnosed him as having VZV myelitis complicated by HZ based on the clinical course and biochemical examination and treated him with intravenous acyclovir (15 mg/kg body weight/day, 10 days) and intravenous methylprednisolone sodium succinate (1000 mg/day, 2 days). On day 5, his lower extremity strength recovered to 3/5 bilaterally and he could walk with a T-cane and assistance. On day 12, his lower limb strength recovered to 4/5 bilaterally and he could walk with a T-cane without assistance. On day 17, he was able to walk independently. On day 22, his lower limb strength recovered to 5/5 bilaterally, and he was discharged without postherpetic neuralgia, recurrence of HZ, or myelitis.

## Discussion

We experienced three cases of aseptic meningitis by VZV and one case of VZV myelitis after HZ dermatitis during treatment with amenamevir for HZ in various immunological backgrounds (Table 1). Table 2 summarizes six HZ cases treated with amenamevir with CNS complications that were previously reported [2-6]. While three cases in our report are regarded as immunocompromised hosts (two cases were taking immunosuppressive drugs, and one case had sigmoid colon cancer as an underlying disease), one case was immunocompetent. All 10 cases were treated with amenamevir against HZ dermatitis before they displayed neurological complications. In all of our cases, VZV DNA in CSF was detected by PCR. According to Nagel et al., the detection rate of VZV DNA in CSF is low (28%) in patients with VZV reactivation and brain vasculopathy, while VZV IgG is highly detected in CSF [7]. The positive PCR analysis in the CSF of the four present cases may have been due to insufficient amenamevir treatment against HZ to exert its antiviral effects in CSF, possibly because of its poor CSF transfer.

**Table 1:** Characteristics of the present cases ^†^VZV DNA was tested by polymerase chain reaction. F: female, iv: intravenous, M: male, MTX: methotrexate, mPSL: methylprednisolone, PSL: prednisolone, RA: rheumatoid arthritis, Th: thoracic spinal cord, VZV: varicella-zoster virus, ACV: acyclovir, V1: first branch of the trigeminal nerve

	Case 1	Case 2	Case 3	Case 4
Age, years	69	64	63	71
Sex	M	F	F	M
Underlying diseases	Sigmoid colon cancer (post-resection)	RA, renal tubeculosis (post-nephrectomy)	RA	Lumbar canal stenosis
Medication on the onset of meningitis/myelitis	None	Salazosulfapyridine (100 mg/day), upadacitinib hydrate (15 mg/day), iguratimod (50 mg/day), and MTX (6 mg/week)	Bucillamine (200 mg/day) and PSL (1 mg/day)	None
Presenting symptoms	Headache	Headache, nausea	Headache	Weakness in both lower limbs
Area of the skin rash	Right V1 nerves	Left V1 nerve	Left Th4-Th10 nerves	Left V1-V2 nerves
Type of CNS complication	Meningitis	Meningitis	Meningitis	Myelitis
CSF test results				
Mononuclear cells, /μL	21	9	492	42
Protein, mg/dL	87	50	174	104
Glucose, mg/dL	54	63	44	47
VZV DNA^†^	Positive	Positive	Positive	Positive
IgG index	Not tested	Not tested	Not tested	0.81
Oligo clonal band	Not tested	Not tested	Not tested	Positive
Opening pressure, mmH2O	180	210	150	160
Culture	Negative	Negative	Negative	Negative
Treatment	ACV (iv)	ACV (iv)	ACV (iv)	ACV (iv), mPSL pulse (iv)
Outcome	Recovered, residual pain on the surface of head	Fully recovered	Fully recovered	Fully recovered

**Table 2:** Characteristics of the previous cases of CNS complications in patients with herpes zoster initially treated by amenamevir ^†^VZV DNA was tested by polymerase chain reaction. AA: aplastic anemia, AAA: abdominal aortic aneurysm, ACV: acyclovir, CKD: chronic kidney disease, CNS: central nervous system, CSF: cerebrospinal fluid, F: female, HT: hypertension, IP: interstitial pneumonia, iv: intravenous, IVIg: intravenous immunoglobulin, M: male, mPSL: methylprednisolone, ND: not described, PSL: prednisolone, VZV: varicella zoster virus

	Case 1	Case 2	Case 3	Case 4	Case 5	Case 6
Age, years	76	72	78	91	79	77
Sex	F	M	F	M	F	F
Underlying diseases	IP	AA, HT, gastric cancer (gastrectomy)	HT, breast cancer (mastectomy)	AAA, CKD	Rheumatoid arthritis	Gastric cancer (post-surgery)
Medication on the onset of meningitis/myelitis	PSL 10 mg/day	ND	ND	ND	PSL, MTX	None
Presenting symptoms	Ascending quadriplegia, cognitive impairment	Right facial muscle weakness, diplopia	Fever, unconsciousness, left hemiplegia	Fever, headache, neck stiffness, decreased visual acuity in the left eye	Opthalmoplegia	Weakness in the left upper limb
Area of the skin rash	Right C7-Th1 nerves	Right shoulder to upper arm	Right V1 nerve	Left V1 nerve	Right V1	Left C6
Type of CNS complication	Polyradiculoneuritis (spinal root of C7-Th1 and L4-S2)	Multiple intracerebral hemorrhages due to vasculopathy, facial and oculomotor nerve palsy	Meningoencephalitis, vasculitis	Meningitis	Meningitis, opthalmoplegia	ND
CSF test results						
Mononuclear cells, /μL	13	97	25	17	12	ND
Protein, mg/dL	73	178	72	144	66.1	ND
Glucose, mg/dL	81	69	60	68	62	ND
VZV DNA^†^	Negative	Negative	Not tested	Negative	Negative	ND
IgG index	ND	ND	ND	ND	ND	ND
Oligo clonal band	ND	ND	ND	ND	ND	ND
Opening pressure, mmH_2_O	170	ND	ND	160	ND	ND
Culture	Negative	Negative	Negative	Negative	ND	ND
Treatment	ACV, IVIg	ACV, mPSL pulse	ACV, steroid pulse	ACV	ACV, oral PSL	ACV, mPSL (iv)
Outcome	Recovered, requiring rehabilitation	Recovered, residual facial nerve palsy	Recovered, residual disturbance of consciousness	Recovered, requiring rehabilitation	Recovered	Improved
Reference	[2]	[3]	[4]	[5]	[6]	[6]

Amenamevir is an anti-herpes drug with a novel mechanism of action targeting the helicase-primase complex [8]. The current indication of amenamevir is only for HZ dermatitis, but not for CNS complications. Animal studies have shown that the amenamevir concentration in the CNS of mice is 1/10 of that in serum, suggesting low CSF transfer in humans [9]. We speculate that the current standard amenamevir dosing cannot achieve adequate CSF concentrations. On the other hand, the intravenous administration of acyclovir produces CSF concentrations as high as approximately half the serum concentration [10].

Acyclovir has been observed to inhibit the biosynthesis of viral DNA while the host's immune system ultimately eliminates the virus. In Cases 2 and 3, the patient was treated with PSL, MTX, and upadacitinib for treating RA. According to the previous research, it has been found that PSL can suppress interleukin (IL) gene expression by forming heterodimers with transcription factors such as activator protein 1 (AP-1) and nuclear factor-kappa B (NF-kB), which mediate inflammatory responses [11]. Additionally, MTX has been reported to reduce IL-6, soluble IL-2 receptors, soluble tumor necrosis factor (TNF) receptors, and IL-8 in the blood [12]. Upadacitinib is a selective Janus kinase (JAK) inhibitor that interferes with the ATP-binding site of JAKs, resulting in the suppression of downstream signaling pathways, which can have immunomodulatory effects by reducing interferon gamma (IFN-γ), IL-15, and IL-21 [13]. It is possible that the reduced innate and acquired immunity due to suppressed cytokine production could potentially affect viral replication and lead to CNS infection with VZV in the present cases. Therefore, it may be worth considering the possibility that patients taking immunosuppressive drugs or with compromised immune function due to diseases such as RA, as in these cases, may have an increased risk of developing CNS infections caused by VZV. It is important to closely monitor their clinical and CSF findings, as they may deteriorate early in the disease. Additionally, it is recommended to administer acyclovir, which is highly CSF-transferable, in such patients from the beginning of ΗΖ treatment.

Upadacitinib is approved by the US Food and Drug Administration, the European Medicines Agency, as well as other agencies around the world for the treatment of several chronic inflammatory diseases, including RA [14]. Winthrop et al. revealed that RA patients from Asia and those with a history of HZ may be at an increased risk of HZ while receiving upadacitinib [15]. Importantly, in their pooled analysis of six phase III clinical trials, one case of VZV meningitis patient (a 64-year-old male Japanese) was reported out of 4413 RA patients receiving upadacitinib who developed HZ. in contrast, none developed VZV meningitis out of 893 RA patients receiving MTX with or without adalimumab. Although statistically non-significant, we speculate that RA patients receiving upadacitinib might be at a higher risk of developing VZV meningitis when they suffer from HZ, and think that special attention should be given to the choice of drugs when treating HZ patients with RA receiving upadacitinib to treat the patient effectively.

It has been reported that ocular herpes zoster is associated with a high risk of developing CNS complications such as meningitis [16]. Indeed, in our Case 1 there was no obvious ocular herpes zoster, but there were findings suggestive of ocular herpes zoster, such as increased intraocular pressure and decreased corneal sensitivity. In such cases, where there is a higher risk of CNS complications by HZ, we advise that acyclovir, which is more likely to be transferred to the CSF than amenamevir, should be used from the beginning of treatment of HZ.

In the outpatient setting, amenamevir may be a more frequent prescription choice, especially for elderly patients with HZ, as no volume adjustment based on creatinine clearance is necessary. However, for immunocompromised patients, such as those taking immunosuppressive medications, or for ocular HZ patients at a high risk of CNS complications, as in the present cases, it may be preferable to use acyclovir or valacyclovir to reduce the risk of complications [5]. Additionally, Skripuletz et al. conducted a study on VZV CNS complications and found that cardiovascular diseases were the most common comorbidity, followed by impaired renal function [17]. Therefore, it may be advisable to consider using acyclovir or valacyclovir instead of amenamevir to manage CNS complications, especially in the presence of these comorbidities.

Typically, the diagnostic criteria for infectious myelitis include abnormalities on spinal cord MRI [18]. In contrast, in a previous report summarizing the characteristics of myelitis caused by VZV, six of 24 cases had no obvious abnormality on the spinal cord MRI [19]. In our Case 4, the non-gadolinium contrast-enhanced spinal cord MRI in the acute phase was normal. Still, the clinical presentation and spinal fluid findings led to the diagnosis that the patient had myelitis. It remains possible that the gadolinium contrast-enhanced MRI of the spinal cord in the acute phase, or MRI studies that were repeated later could have detected the abnormality.

Myelitis due to VZV reactivation is a rare complication that can occur in both immunocompromised and immunocompetent patients [20]. While VZV myelitis may occur without typical skin lesions and at different levels in immunocompromised patients, it typically presents with dermatomal rashes followed by myelitis at the corresponding level in immunocompetent patients [19-21]. Despite these differences, good outcomes are generally observed in both patient populations. Regarding the fourth case in our report, it is worth noting that the patient presented with an atypical symptomatology, including paraplegia and eruptions in the first and second branches of the left trigeminal nerve on the face despite being immunocompetent. As we have observed, patients with HZ who are immunocompetent may exhibit unusual symptoms when they develop CNS complications.

## Conclusions

The present report described four cases of aseptic meningitis and viral myelitis caused by VZV following amenamevir treatment against HZ with positive VZV DNA in CSF revealed by PCR. Because amenamevir has poor CSF transfer, it should be avoided in patients at high risk of VZV CNS complications, such as those on immunosuppressive drugs. Drugs such as acyclovir, which has higher CSF transfer, should be preferably used in these clinical conditions.
